# Supplementary figures and images for: School and home-based educational intervention in urban Kenya: Sustained improvements in knowledge, attitudes, and practices for Aedes aegypti control
Source: PLOS Glob Public Health. 2025 Jun 4;5(6):e0004567. doi: 10.1371/journal.pgph.0004567 (PMC12136349; doi:10.1371/journal.pgph.0004567)

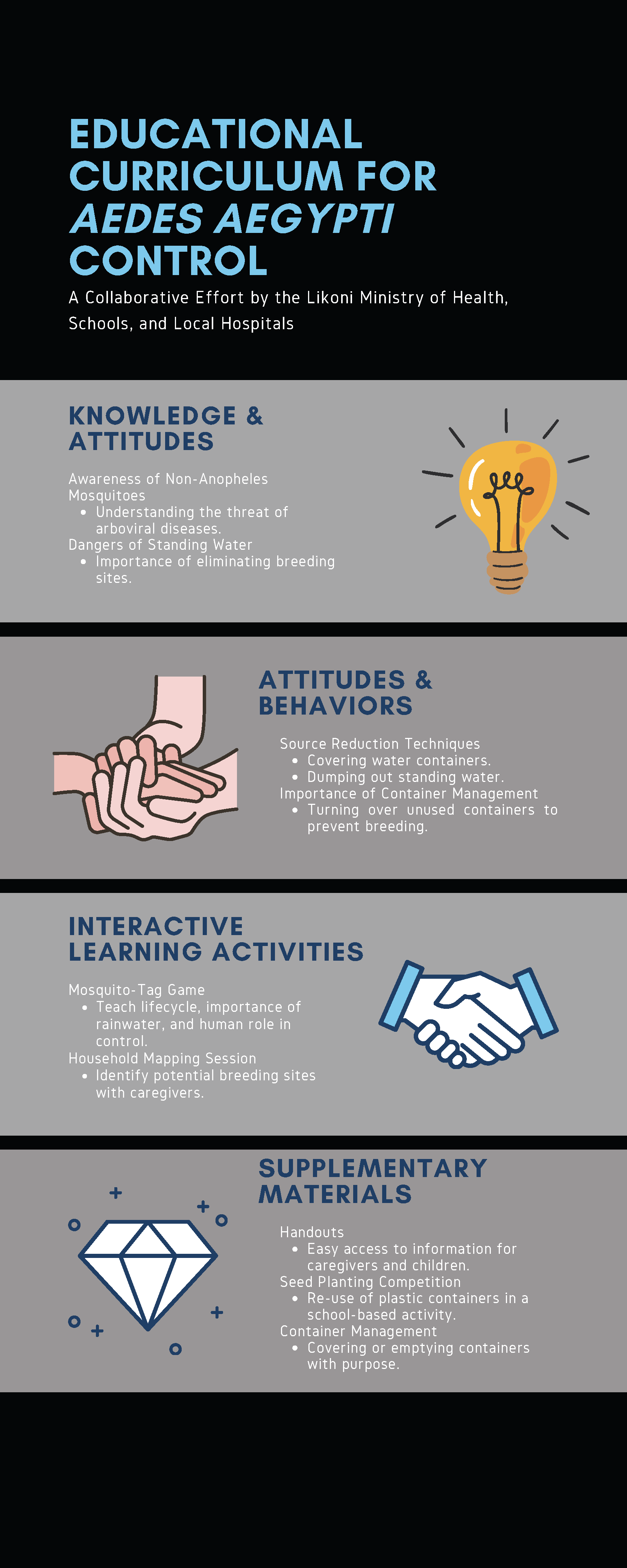

Supplement: S1 Fig — This infographic summarizes the key educational messages related to vector-borne disease prevention, environmental sanitation, personal hygiene, and community engagement. (TIFF) [file pgph.0004567.s001.tiff]

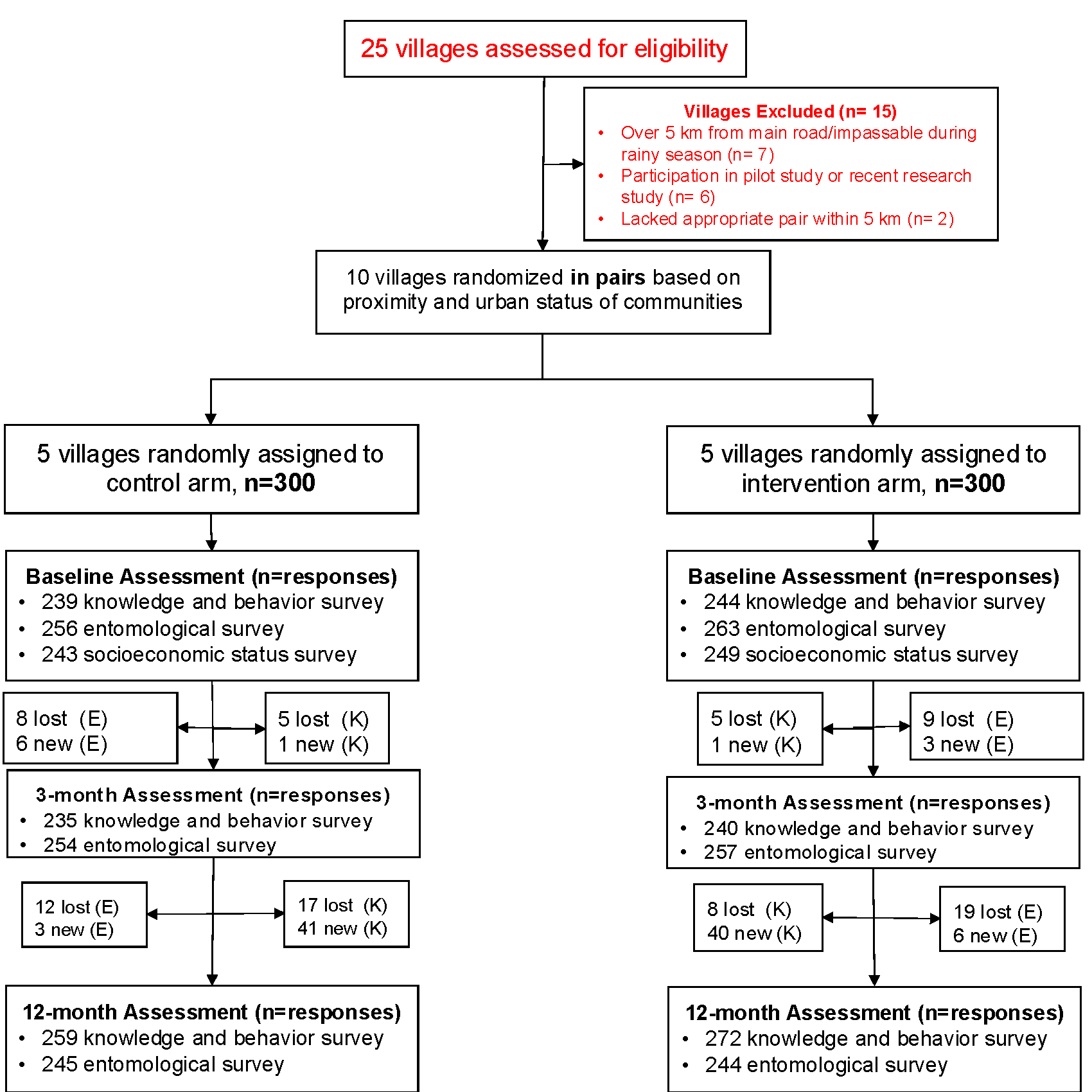

Supplement: S2 Fig — Flowchart describing the number of caregivers included at each stage of the analysis. Exclusion and attrition numbers at each stage are detailed. (TIFF) [file pgph.0004567.s002.tiff]

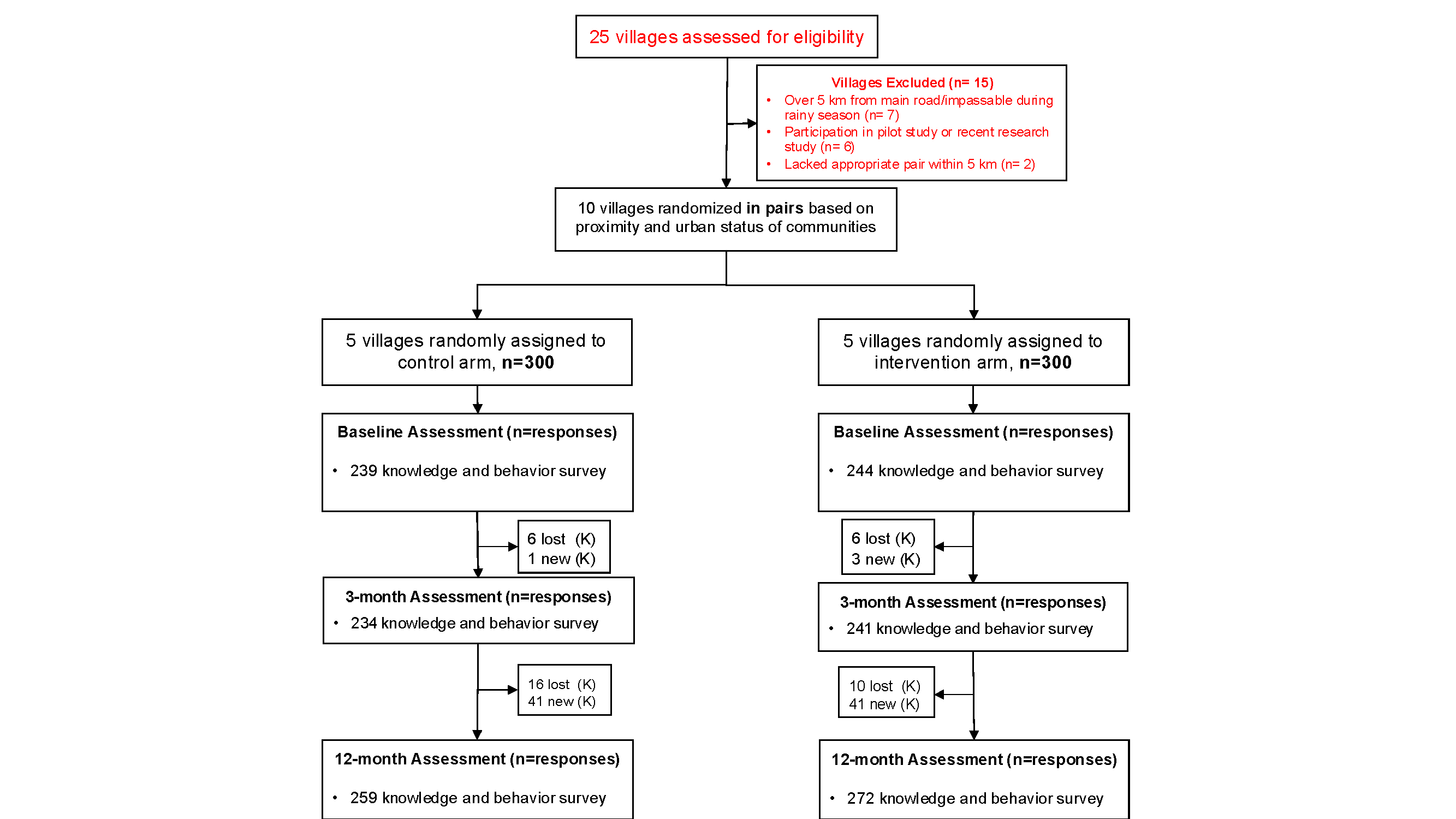

Supplement: S3 Fig — Flowchart describing the number of children included at each stage of the analysis. Exclusion and attrition numbers at each stage are detailed. (TIFF) [file pgph.0004567.s003.tiff]
