# Supplementary material for: School and home-based educational intervention in urban Kenya: Sustained improvements in knowledge, attitudes, and practices for Aedes aegypti control
Source: PLOS Glob Public Health. 2025 Jun 4;5(6):e0004567. doi: 10.1371/journal.pgph.0004567 (PMC12136349; doi:10.1371/journal.pgph.0004567)
Supplement: S1 Text — (DOCX) [file pgph.0004567.s005.docx]

Inclusivity in global research

PLOS’ policy on inclusivity in global research aims to improve transparency in the reporting of research performed outside of researchers’ own country or community and ensures that PLOS publications reporting global research adhere to high standards for research ethics and authorship. Authors of relevant research articles may be asked to complete the questionnaire below, which outlines ethical, cultural, and scientific considerations specific to inclusivity in global research. This questionnaire may be requested when researchers have travelled to a different country to conduct research, if research uses samples collected in another country, research with Indigenous populations or their lands, or if research is on cultural artefacts. Researchers travelling to another country solely to use laboratory equipment will not normally be required to complete the questionnaire. However, the questionnaire can be requested at the journal’s discretion for any submission – if you have been requested to complete this questionnaire by the PLOS journal you submitted to, please do so.

Please complete the questionnaire below and include this as a Supporting Information file with your manuscript. Note that if your paper is accepted for publication, this checklist will be published with your article in the supporting information files. Please ensure that you reference the checklist in the main body of your manuscript. We suggest adding a subsection ‘Inclusivity in global research’ to your Methods section and adding the following sentence: “Additional information regarding the ethical, cultural, and scientific considerations specific to inclusivity in global research is included in the Supporting Information (SX Checklist)”

The questions have been designed to be applicable to a wide range of study types, and there are subsections for both human subjects research and non-human subjects research. If any of the questions are not relevant to your research please mark them as “N/A” as appropriate.

**Ethical considerations, permits and authorship**

*This section is applicable to all research types.*

Provide details as to who granted permissions and/or consent for the study to take place in the Methods section of your manuscript. This should include the names of **all** ethics boards, governmental organizations, community leaders or other bodies that provided approval for the study. If individuals provided approval refer to these people by their role or title but do not list their name(s).

Reported on page number: 7 (Lines 337-339) and 10 (Lines 487-491)

If there were any deviations from the study protocol after approval was obtained please provide details of these changes in the Methods section of your manuscript.
Did this study involve local collaborators that are residents of the country where the research was conducted or members of the community studied? If you do not have any authors from said communities, please provide an explanation for this below.

Reported on page number: NA

Yes. Francis Mutuku, Gladys Agola, and Mwashee Lutt are all members of the community in Kenya and helped coordinate study efforts. They have all been listed as authors due to their contribution to developing the project and collecting data.

Everyone listed as an author should meet PLOS’ criteria for authorship and all individuals who meet these criteria should be included in the author byline, rather than the acknowledgements. For further information please see the journal’s Authorship Policy.

**Human subjects research (e.g. health research, medical research, cross-cultural psychology)**

Did you obtain written informed consent from a representative of the local community or region before the research took place? How did you establish who speaks for the community? Details of written informed consent obtained from study participants should be reported separately in the Methods section of your manuscript.

Yes, written informed consent was obtained from a representative of the local community before the research took place. We collaborated with the Likoni Ministry of Health (MOH) and the MOH vector-borne disease control unit to ensure community approval and engagement. Additionally, written informed consent was separately obtained from adult caregivers for both their participation and that of the enrolled children, as detailed in the Methods section.

How did members of the local community provide input on the aims of the research investigation, its methodology, and its anticipated outcome(s)?

A curriculum committee was formed, including representatives from the Likoni MOH, MOH vector-borne disease control unit, school teachers, and local hospitals. Their insights ensured that the curriculum was culturally appropriate and addressed community-relevant health concerns. We also used feedback from a pilot study involving a smaller sample of households and schools to refine the intervention before full-scale implementation.

When engaging with the local community, how did you ensure that the informed consent documents and other materials could be understood by local stakeholders?

All informed consent documents and educational materials were provided in the local language spoken by the community. The research team members conducting the intervention and obtaining consent were fluent in the local language, enabling effective communication. Also, informed consent procedures were conducted orally in cases where literacy might have been a barrier, with the opportunity for participants to ask questions and seek clarification before signing the consent forms.

Will the findings of the research be made available in an understandable format to stakeholders in the community where the study was conducted (e.g. via a presentation, summary report, copies of publications, etc.)? Please provide details of how this will be achieved.

A summary report has already been provided to key stakeholders, including children, teachers, and members of the Ministry of Health. Also, educational materials developed for the intervention were distributed to participating schools and caregivers (including controls). We also plan to share copies of publications with community representatives and explore opportunities for further dissemination through local health organizations and schools.

**Non-human subjects research using specimens/ animals collected as part of the study, or those housed in archival collections. Examples include archaeology, paleontology, botany and zoology.**

Did the permission you obtained from a local authority to perform the study include an agreement on access to outputs and benefit sharing? This may include procedures to enable fair distribution of the benefits and resources arising from the research performed. Please include any details of Prior Informed Consent and Benefit Sharing Agreements obtained. These may be required by field-specific regulations, for example the Convention on Biological Diversity (CBD) and the associated Nagoya Protocol.

NA

If the material used in your study was imported, please A) provide the year it was imported and B) indicate whether permits were obtained to import/export the materials used, C) provide details of any permits obtained. If this information is not available, please indicate this.

NA

If you used archival specimens, please state how the material used in your study was acquired by the institute it is held in and provide details of any permits obtained for the original excavations/ sample collection. If this information is not available, please indicate this.

NA

How was the potential cultural significance of the materials collected in your study to local communities considered in your research design? Were Indigenous peoples and/or local researchers and institutions involved with archaeological excavations / collection of specimens? If so, please provide a description of their involvement.

NA

If your manuscript includes photographs of human remains please indicate whether authors obtained permission from descendants or affiliated cultural communities to do so.

NA
