# Supplementary material for: School and home-based educational intervention in urban Kenya: Sustained improvements in knowledge, attitudes, and practices for Aedes aegypti control
Source: PLOS Glob Public Health. 2025 Jun 4;5(6):e0004567. doi: 10.1371/journal.pgph.0004567 (PMC12136349; doi:10.1371/journal.pgph.0004567)
Supplement: Attachment — Submitted filename: Response to Reviewers.docx [file pgph.0004567.s007.docx]

**Response to Reviewers**

**Manuscript Title:** School and Home-Based Educational Intervention in Urban Kenya: Sustained Improvements in Knowledge, Attitudes, and Behaviors for Aedes aegypti Control

**Authors:** Prathik Kalva, Jenna Forsyth, Francis Mutuku, Gladys Agola, Mwashee Lutt, A. Desiree LaBeaud

**Journal:** PLOS Neglected Tropical Diseases

**Dear Editor and Reviewers,**

We would like to thank you for the thorough review of our manuscript and for providing valuable feedback. We have carefully considered each comment and have made the necessary revisions to improve the manuscript. Below, we provide detailed responses to each point raised by the reviewers.

**Reviewer #1:**

**General Comment:**

- **Comment:** The authors reported an important topic of Aedes aegypti control. It’s a caregiver-targeted household-based intervention combined with a school-based educational intervention to build knowledge, skills, and create a sense of self-efficacy in a dengue-endemic area of urban Kenya. The study is well designed, but the review has some concerns on the data reported.

**Response:** Thank you for your positive feedback on the importance and design of our study.

**Major Comment:**

- **Comment:** The authors reported the detail KAB of children as “agents of change” and caregivers’ responses to the questionnaires were not analysed due to differences at baseline. The reviewer is not clear what is meant by the “differences at baseline” and how was that assessed. I propose the authors consult a statistician how to analyse KAB of children as well as caregivers for the entomological indices as outcome.

**Response:** We have clarified what we meant by differences at baseline and consulted a statistician. The phrase "differences at baseline" refers to the initial variations in the KAP characteristics between the intervention and control groups with the caregivers. We have included this clarification in the Results section (Lines 556-559). This data can be found in Table S1 of the Supplement.

**Detailed Comments:**

- **Introduction:**
  - **Comment:** Reference 25 and 26 are repeated. Please cite the correct paper for Ref 26.
    - **Response:** We have corrected the references and ensured that this reference is only listed once.
- **Methods:**
  - **Comment:** Please briefly mention why Likoni was selected and if available, provide dengue prevalence data.
    - **Response:** We have added a brief explanation for selecting Likoni and provided dengue prevalence data in the Methods section (Lines 257-260).
  - **Comment:** Expand the description on pilot, rather than simply citing reference 24.
    - **Response:** We have expanded the description of the pilot study to provide more details on its purpose and outcomes (Lines 272-276).
  - **Comment:** Villages were selected. Please add a statement, why they are considered as Urban communities?
    - **Response:** We have added a statement explaining why the selected villages are considered urban communities (Lines 280-281).
  - **Comment:** Add about ‘Fourth grade student’ this section. Currently, Fourth grade was mentioned in abstract and results only.
    - **Response:** We have included information about fourth-grade students in the Methods section (Lines 293-297).
  - **Comment:** Are there any implications for recruiting less/ analyzing less than the intended numbers?
    - **Response:** We have addressed the implications of recruiting fewer participants than intended in the Methods section (Lines 481-483).
  - **Comment:** Were there any differences in the characteristics of the children who completed the survey and who did not?
    - **Response:** Since we do not have data on the children who did not complete the initial survey, we are unable to include this information in the manuscript.
  - **Comment:** If possible, add Consort flow chart for clustered RCTs.
    - **Response:** We have added our consort flow chart in the Supplement. Please keep in mind that these numbers reflect the total responses at each time point, but for analysis we only included participants for which we had data at every time point (Lines 288-289).
  - **Comment:** How many trainers were there in home training sessions? Do they speak the same language as the caregivers?
    - **Response:** We have provided details about the number of trainers and their language proficiency in the Methods section (Lines 329-333).
  - **Comment:** Describe how the scoring of the KAB were done?
    - **Response:** We have described the scoring of the KAB surveys in the Methods section (Lines 399-411 and 434-451).
- **Results:**
  - **Comment:** Please confirm the numbers of caregivers were involved in data collection and the number used data analysis are the same (i.e., 492). If any caregivers were excluded because of missing data, please edit the paragraph accordingly.
    - **Response:** We have confirmed the numbers of caregivers involved in data collection and analysis and edited the paragraph accordingly (Lines 502-505).
  - **Comment:** Similarly, please describe how many children were recruited and re-confirm how many children were analyzed (i.e., 445).
    - **Response:** We have confirmed the number of children recruited and analyzed and included this information in the Results section (Lines 534-536).
- **Table 1:**
  - **Comment:** Table 1 shown for the numbers of caregivers (i.e., 243 vs 249). If the main intention was to report KAB of children, will authors consider to report the Table 1 by the numbers of children instead (i.e., 217 vs 228)?
    - **Response:** Thank you for your suggestion. We included the whole cohort for robustness and to provide a comprehensive overview of the study population. The caregiver information is important because caregivers play a crucial role in the household environment and their characteristics can influence the effectiveness of the intervention. By including both caregivers and children, we can better understand the dynamics within the household and how they impact knowledge, attitudes, and behaviors related to Aedes aegypti control. Additionally, reporting the numbers of caregivers ensures transparency and allows for a more detailed analysis of the intervention's impact on the entire household.
  - **Comment:** Were there any statistical tests performed to assess any differences in the two groups?
    - **Response:** We have added p-values to assess for any differences in the two groups (Table 1).
  - **Comment:** Numbers were missing for female and marital statuses.
    - **Response:** Thank you for pointing this out. We have went in and fixed this (Table 1).
- **Table 2:**
  - **Comment:** Table 2, add p-values comparing baseline data.
    - **Response:** We have added p-values comparing baseline data in Table 2.
  - **Comment:** Edit the table title to reflect the reporting of Entomological indices in Table 2.
    - **Response:** We have edited the table title to reflect the reporting of entomological indices.
- **Table 3:**
  - **Comment:** Table 3 is missing in the submission/ review.
    - **Response:** We have went in and added Table 3 to the submission. Thank you for pointing this out.
- **Discussion:**
  - **Comment:** Add reference for third and fifth paragraphs (i.e., The house index… Overall…).
    - **Response:** Thank you for your feedback. We have added references to the third and fifth paragraphs in the Discussion section. In the third paragraph, we now cite a study that discusses the influence of seasonal rainfall and the resilience of mosquito breeding sites, supporting our explanation for the observed increase in the house index. In the fifth paragraph, we have incorporated a reference that addresses the adoption of source reduction behaviors and the importance of aligning interventions with existing community practices to enhance effectiveness. These references provide additional context and support for our findings (Lines 708, 746, 751)
  - **Comment:** Entomological indices supposed to validate the self-reported measure. Therefore, self-reported measure should not be listed as a limitation.
    - **Response:** We have clarified the role of entomological indices in validating self-reported measures and ensured self-reported measures are not listed as a limitation (Lines 765-774).
- **Conclusion:**
  - **Comment:** The second sentence was not supported by the findings from the study (i.e., The findings underscore ….). Please revise.
    - **Response:** We have rewritten the conclusion to address this and only included findings that are supported by the study (Lines 778-812).
  - **Comment:** Conclusions should not introduce new agenda. Please clarify why the need for solid waste management was mentioned in conclusion?
    - **Response:** We have rewritten our conclusion and removed this from the section (Lines 778-812).

**Reviewer #2:**

**General Comment:**

- **Comment:** Lastly, and most importantly, the authors should clearly demonstrate the novel findings of their study in the body of literature. This could be achieved by highlighting how their study adds to existing literature and identifying any new or significant findings.

**Response:** We have highlighted the novel findings of our study and how it adds to the existing literature in the Conclusion section (Lines 778-784).

**Introduction:**

- **Comment:** When it comes to a review of the literature, the introduction is quite brief. Information about dengue and other arboviruses' morbidity or mortality, their prevalence globally or in Sub-Saharan Africa, who is more impacted, and why should be included. The authors may take advantage of the extensive body of research on arboviruses and the role children tackle them. The authors might offer a list of additional potential interventions in addition to school-based. Lastly, other than examining knowledge, attitudes, or behavioral changes, what are the specific guiding research questions/hypotheses for this study?
  - **Response:** We have expanded the introduction to include information about dengue and other arboviruses' morbidity and mortality, their global prevalence, and their impact in Sub-Saharan Africa. We have also included specific guiding research questions and hypotheses for the study (Lines 81-103 and 236-241).

**Methods:**

- **Comment:** I am unclear why the authors included children in the KAP assessment. Also, not clear how the questions administered were standardized and validated? Authors need to define what increases or decrease in knowledge, attitude, or behavioral change. How were these variables measured? Analysis should consider additional statistical tests beyond descriptive statistics.
  - **Response:** We have clarified the inclusion of children in the KAP assessment and described how the questions were standardized and validated. We have defined the variables and included additional statistical tests (chi-squared and correlational) beyond descriptive statistics in the Methods section and included data in the Results section (Lines 399-457, 547-554, 618-621 (new table), 639-644, 663-667).

We hope that the revisions and responses provided address the concerns raised by the reviewers. We believe that these changes have strengthened the manuscript and we look forward to your feedback.

Sincerely,

Prathik Kalva
[kalvajr@gmail.com](mailto:kalvajr@gmail.com) | Prathik.kalva@bcm.edu
