# Supplementary material for: School and home-based educational intervention in urban Kenya: Sustained improvements in knowledge, attitudes, and practices for Aedes aegypti control
Source: PLOS Glob Public Health. 2025 Jun 4;5(6):e0004567. doi: 10.1371/journal.pgph.0004567 (PMC12136349; doi:10.1371/journal.pgph.0004567)
Supplement: Attachment — Submitted filename: Response_to_Reviewers_auresp_2.docx [file pgph.0004567.s008.docx]

**Response to Reviewers**

**Manuscript Title:** School and Home-Based Educational Intervention in Urban Kenya: Sustained Improvements in Knowledge, Attitudes, and Behaviors for Aedes aegypti Control

**Authors:** Prathik Kalva, Jenna Forsyth, Francis Mutuku, Gladys Agola, Mwashee Lutt, A. Desiree LaBeaud

**Journal:** PLOS Global Public Health

**Dear Editor and Reviewers,**

We would like to thank you for the thorough review of our manuscript and for providing valuable feedback. We have carefully considered each comment and have made the necessary revisions to improve the manuscript. Below, we provide detailed responses to each point raised by the reviewers.

**Reviewer Comments:**

"Line 40: missing p value in parentheses

- We have went in and added the p-value for this (Line 39). I am not sure if the line numbers are different, so please let me know if there is somewhere else that needs changing.

Line 569: ‘at 12 months’ not ‘as 12 months’”

- We have went in and changed this (Lines 511 and 516).

We hope that the revisions and responses provided address the concerns raised by the reviewers. We believe that these changes have strengthened the manuscript and we look forward to your feedback.

Sincerely,

Prathik Kalva
[kalvajr@gmail.com](mailto:kalvajr@gmail.com) | Prathik.kalva@bcm.edu
